# Time to death in breast cancer patients as an indicator of treatment response

**DOI:** 10.1007/s10549-018-4935-3

**Published:** 2018-08-30

**Authors:** Steven A. Narod, Vasily Giannakeas, Victoria Sopik

**Affiliations:** 1 Women’s College Research Institute, Women’s College Hospital, 76 Grenville Street, Toronto, ON M5S 1B2 Canada; 2 Dalla Lana School of Public Health, University of Toronto, Toronto, ON Canada

**Keywords:** Breast cancer, Mortality, Survival, Progression

## Abstract

**Purpose:**

To describe the mortality experience of women who die of breast cancer in the 20-year period post-diagnosis using various metrics, including annual mortality rates, Kaplan–Meier survival curves and time-to-death histograms.

**Methods:**

We generated three visual representations of SEER-based and hospital-based breast cancer patient cohorts using three different metrics of mortality.

**Results:**

The greatest impact of most prognostic factors was on the probability of latent metastases present after treatment, but for some factors the primary impact was on the time to death for those women with metastases.

**Conclusions:**

The use of time-to-death statistics to display mortality benefits for treated versus untreated women helps facilitate the distinction between treatments which increase the likelihood of cure and treatments that delay cancer growth.

**Electronic supplementary material:**

The online version of this article (10.1007/s10549-018-4935-3) contains supplementary material, which is available to authorized users.

## Part 1: Measuring mortality

Approximately 25% of women with breast cancer diagnosed in the United States will die of breast cancer within 20 years, providing they do not die of something else [1, 2]. In the simplest survival model, we assume that women who have latent (occult) distant metastases after surgery and adjuvant chemotherapy are at risk of dying of the cancer. Under this model, the annual mortality rate for a given patient can be predicted by the probability that a cancer patient presents with occult metastases at diagnosis, the probability that the adjuvant treatment has eliminated all the metastases and the distribution of the times to death for the women who eventually die. Life expectancies calculated in this way are based on probabilistic distributions from large databases and do not permit us to predict with accuracy the time of death of a given patient.

**Fig. 1:**
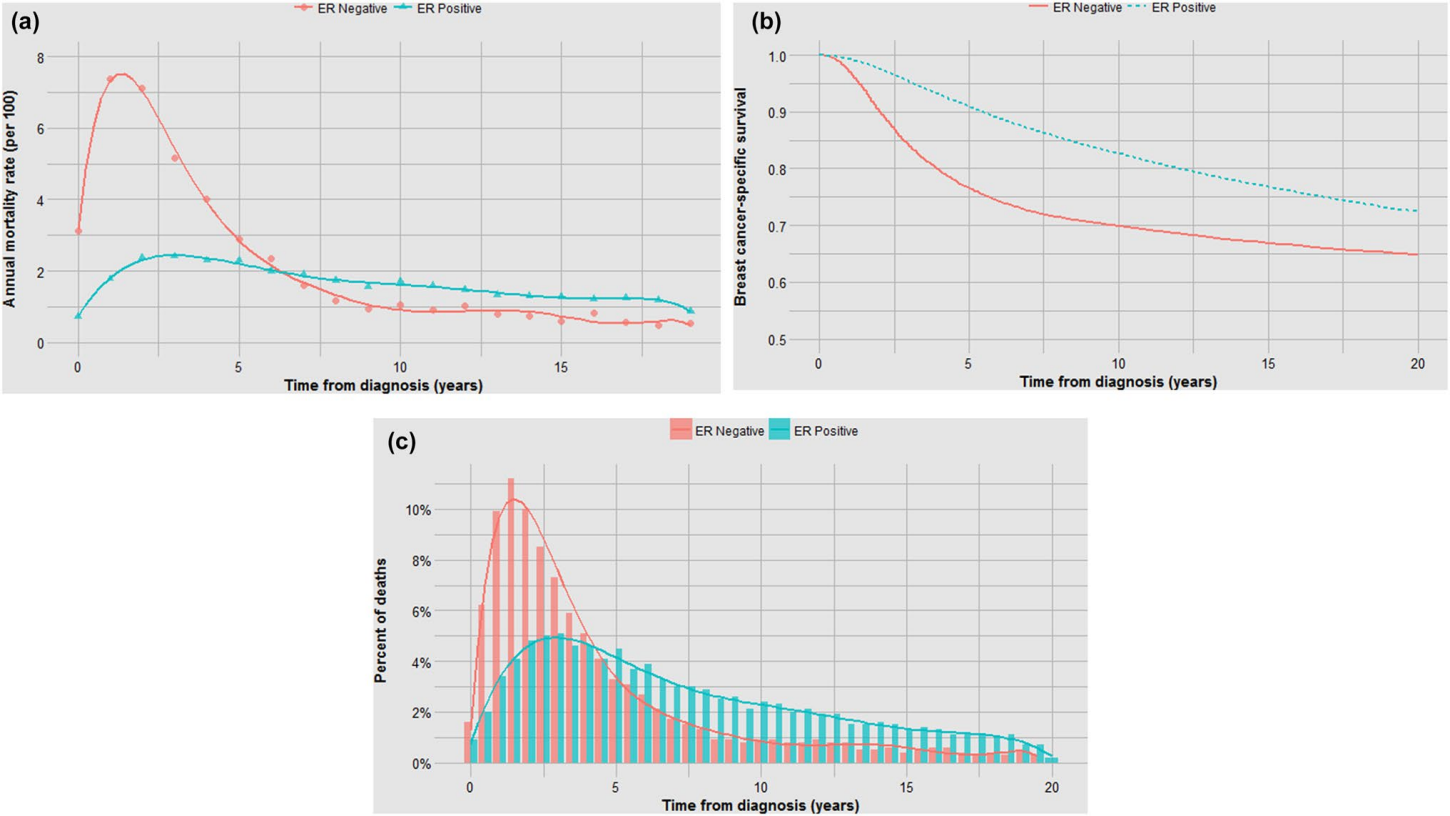
**a** Impact of ER status on annual mortality rates. **b** Impact of ER status on actuarial survival. **c** Impact of ER status on time to death

Prognostic factors might impact on the annual mortality rate through impacting the probability of occult metastases at diagnosis and/or prolonging or accelerating the time to death among women who die (the effect of treatments on these metrics is discussed below). To illustrate, we used the SEER data set to construct mortality curves and time-to-death histograms for various breast cancer patient subgroups. The data set includes 76,173 women diagnosed with invasive breast cancer from 1990 to 1995. In the first example, we compared the mortality experience for ER-positive and ER-negative breast cancer patients. We calculated the annual breast cancer mortality rates for each year from diagnosis for the members of the cohorts from year 1 to year 20. The annual mortality rates (hazard rates) are presented for ER positive and ER negative (stages I–III) in Table 1 (supplemental) and are plotted graphically in Fig. 1a. We generated actuarial survival curves using the Kaplan–Meier method (Fig. 1b). We also plotted the time from diagnosis to death for ER-positive and ER-negative women who died of breast cancer using histograms (Fig. 1c).

**Table 1:** Survival experience: all women with stages I–III breast cancer in the SEER database

Group	*N*	Annual death rate (%)	% of Cohort dead of breast cancer	10-year survival rate (%)	20-year survival rate (%)	Median time to death [10th, 90th percentile]^a^	Mean time to death (years)^a^	% of Breast cancer deaths years 1–5^a^	% of Breast cancer deaths years 15–20^a^
Overall	76,173	1.90	24.2	79.6	71.1	5.0 [1.4, 14.3]	6.5	50.5	8.6
ER negative	14,759	2.63	32.4	69.9	64.9	3.1 [1.1, 10.9]	4.6	70.4	4.2
ER positive	45,647	1.70	22.1	82.6	72.4	6.3 [1.9, 15.3]	7.5	39.3	11.0
Node positive	25,305	4.05	43.5	61.8	49.7	4.4 [1.3, 13.3]	5.9	55.4	6.7
Node negative	50,035	0.99	13.8	89.4	82.8	6.3 [1.8, 15.8]	7.6	40.0	12.1
0.1–1 cm^b^	9,197	0.43	6.50	96.0	91.5	9.1 [2.7, 17.3]	9.4	24.3	19.6
1–2 cm^b^	21,459	0.79	11.4	92.0	85.5	7.8 [2.4, 16.3]	8.5	31.6	14.8
2–5 cm^b^	15,605	1.56	20.1	83.2	74.9	5.5 [1.8, 14.9]	7.0	46.2	9.8
Grade I	7,344	0.65	9.20	94.6	87.3	9.7 [2.9, 17.0]	9.7	23.0	20.5
Grade II	22,585	1.59	21.0	83.9	73.7	6.6 [1.9, 15.6]	7.7	37.9	11.7
Grade III/IV	24,188	2.95	34.4	68.4	61.1	3.8 [1.2, 12.2]	5.2	62.0	5.1
Age ≤ 40	6,225	2.83	39.6	68.6	59.4	4.8 [1.5, 14.3]	6.4	51.3	8.2
Age > 40	69,948	1.81	22.9	80.6	72.3	5.0 [1.3, 14.3]	6.5	50.4	8.6
White	65,013	1.81	23.1	80.6	72.1	5.2 [1.4, 14.5]	6.6	48.9	8.8
Black	6,184	3.25	35.9	67.1	58.3	3.6 [1.1, 12.3]	5.2	63.6	5.9

^a^In deceased patients

^b^Among ER-negative patients only

**Fig. 2:**
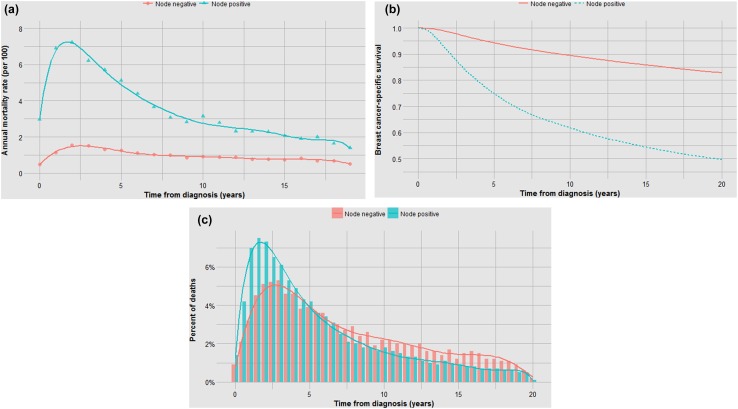
**a** Impact of nodal status on annual mortality rates. **b** Impact of nodal status on actuarial survival. **c**. Impact of nodal status on time to death

**Fig. 3:**
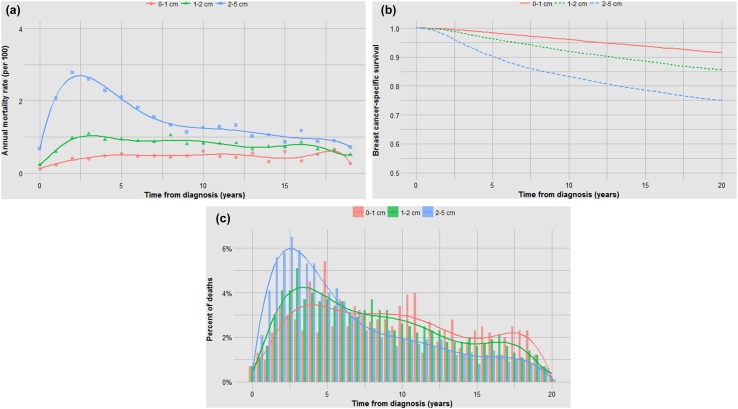
**a** Impact of tumour size on annual mortality rates. **b** Impact of tumour size on actuarial survival. **c** Impact of tumour size on time to death

**Fig. 4:**
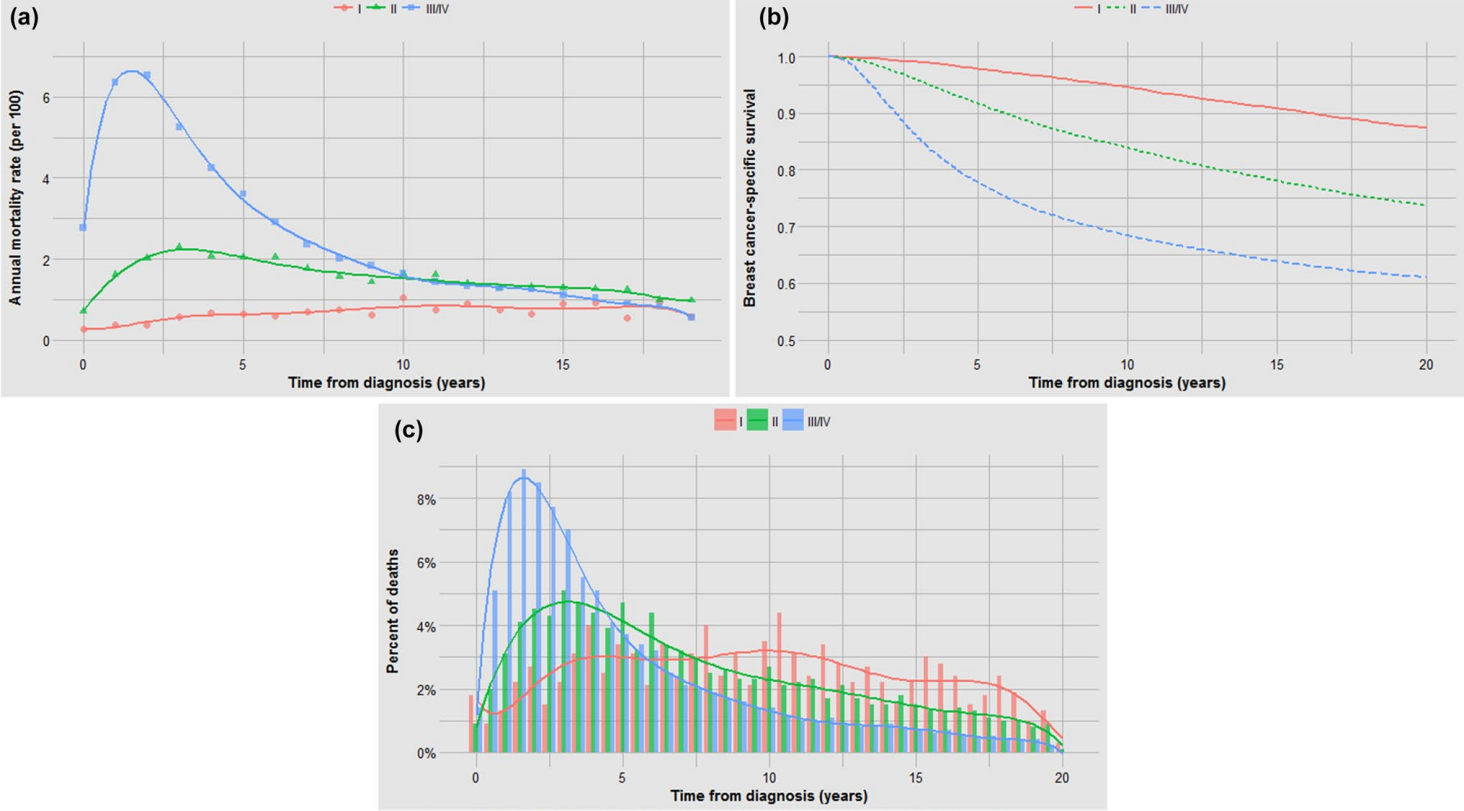
**a** Impact of tumour grade on annual mortality rates. **b** Impact of tumour grade on actuarial survival. **c** Impact of tumour grade on time to death

**Fig. 5:**
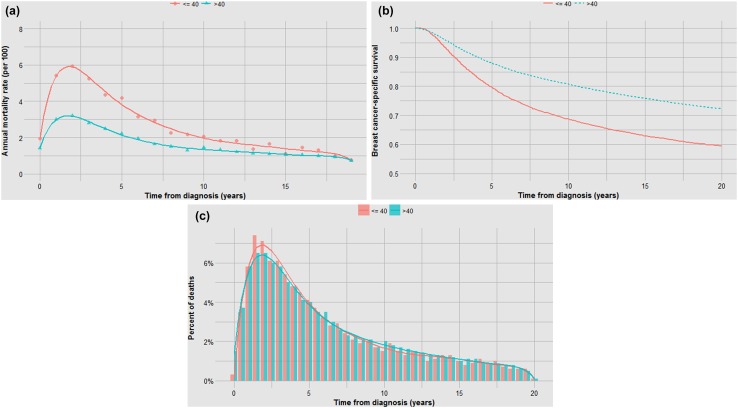
**a** Impact of age at diagnosis on annual mortality rates. **b** Impact of age at diagnosis on actuarial survival. **c** Impact of age of diagnosis on time to death

**Fig. 6:**
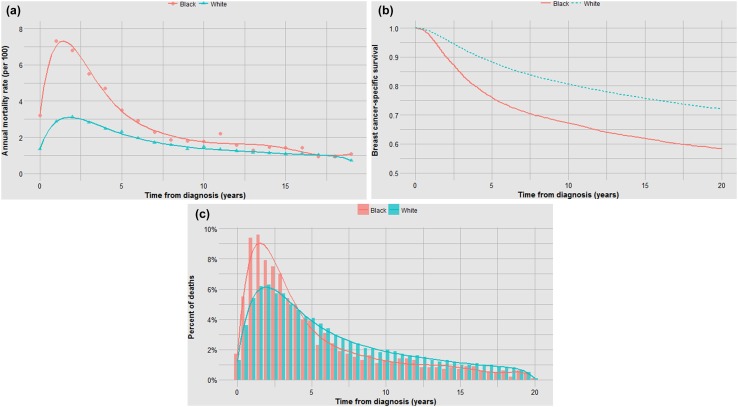
**a** Impact of race on annual mortality rates. **b** Impact of race on actuarial survival. **c** Impact of race on time to death

We repeated these graphs and histograms for patient subgroups defined by nodal status (Fig. 2), by tumour size (0–1 cm, 1–2 cm, 2–5 cm) (Fig. 3), by grade (Fig. 4), by patient age (Fig. 5) and by race (Fig. 6). The data are summarized in Table 1.

In each analysis, survival is compared in three ways—in terms of the annual and cumulative likelihood of death and in the time from diagnosis to death. Different graphical representations are suited to highlight one or another comparison. The data in Fig. 1a, b are inter-changeable in the sense that one can use the data from one to generate the other but the time-to-death data (Fig. 1c) do not allow reconstruction of the other two curves (the denominators are lost).

**Fig. 7:**
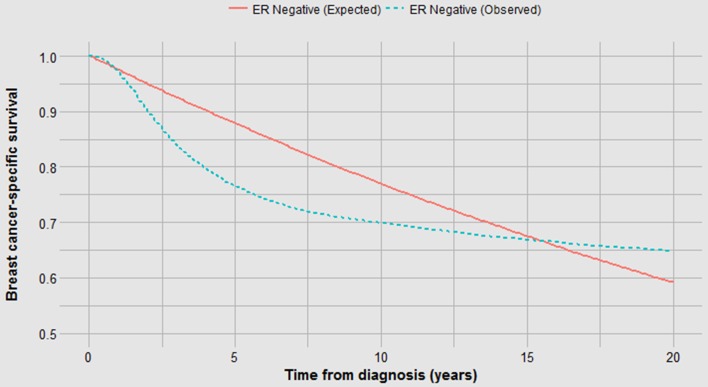
Survival from ER-negative cancer, observed versus exponential (“expected”) decline

Although the annual and cumulative mortality curves are formally inter-changeable, they convey different meanings. The Kaplan–Meier curves are helpful in the sense that they convey the ultimate survival of the patients and predicted survival can be directly read from the *y*-axis. This is relevant to the clinician and the patient. Because of this property, actuarial survival rates are common in clinical research reports, but they may obscure the subtlety associated with changing annual mortality rates. For example, a survival benefit associated with positive ER status is apparent in terms of 20 year survival (73% versus 65%) (Fig. 1b). But it is of interest that the annual mortality of ER-positive cancers exceeds that of ER-negative cancers beyond six years from diagnosis (Fig. 1a). It is also interesting that the annual mortality rates fluctuate greatly for ER-negative cancers—this is a marked departure from exponential decline. Time to death is delayed in ER-positive versus ER-negative cancers by a mean of 2.9 years (Fig. 1c; Table 1). From this, we infer that ER status affects mortality in terms of the likelihood of having metastatic disease at diagnosis as well as the time a cancer takes to become lethal. In Fig. 7, we see that for ER-negative cancer patients deaths accumulate much more quickly than if the annual mortality rate were held constant at 2.6% (Table 1) as opposed to the actual fluctuating rates (supplementary table 1).

**Fig. 8:**
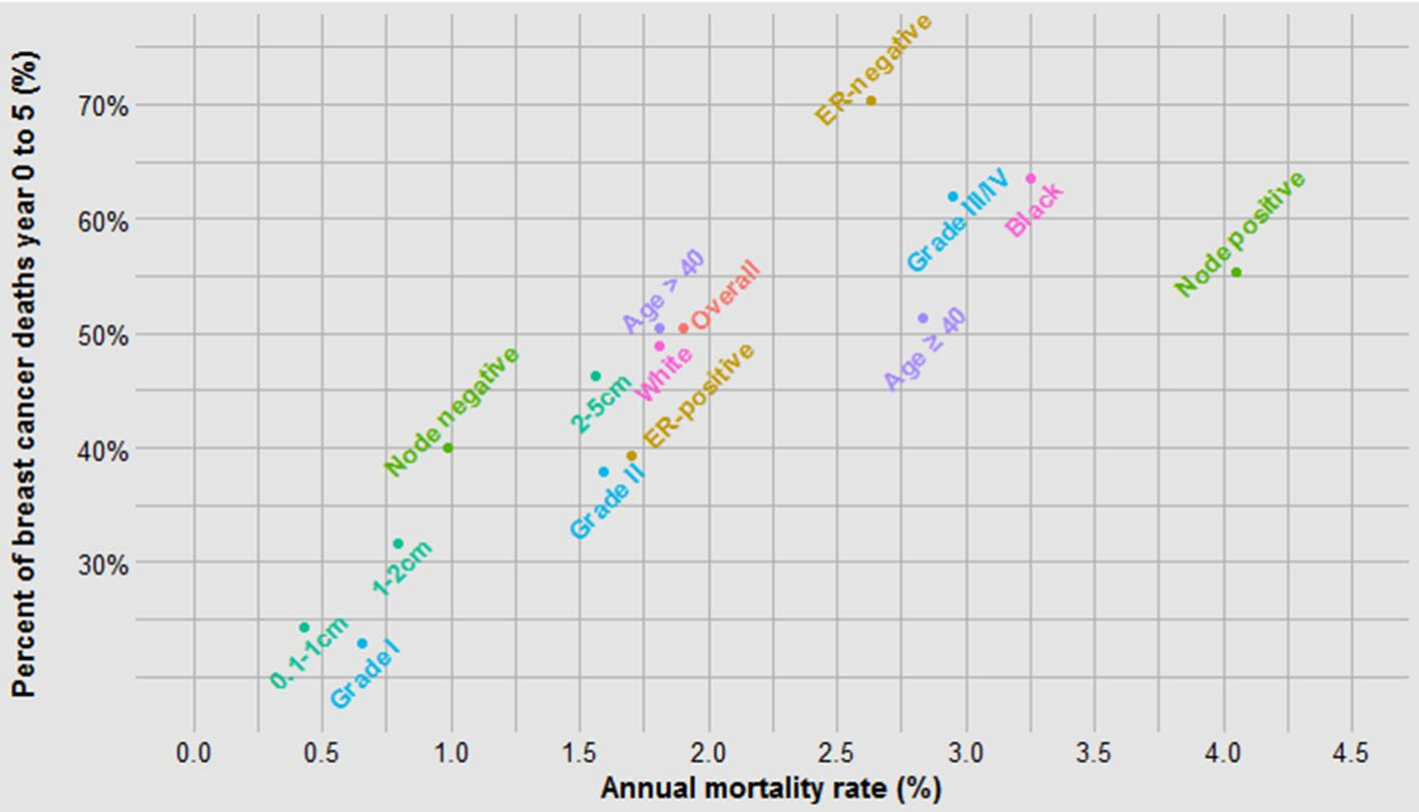
The relationship between annual mortality and the percent of deaths which occur in years 0–5

It is perhaps not surprising that tumour grade predicts both the probability of metastases and the time to death (Fig. 4b, c); what is surprising is that after 15 years the annual mortality rate of a grade I cancer approaches that of a grade III cancer (Fig. 4a). In general, those factors that are associated with high mortality are also associated with a relative high proportion of cancer deaths in the first 5 years and a visible inflection point in risk at approximately 5 years (Figs. 1b, 2b, 3b, 4b, 5b, 6b). These data are summarized in Fig. 8. For factors associated with a low mortality, the annual hazard for death is more or less constant over 20 years and there are no obvious inflections.

Age of diagnosis is singular in that it impacts greatly on annual mortality rates (Fig. 5a) and on ultimate survival (Fig. 5b) but does not influence time to death (Fig. 5c). From this, we infer that a young woman with breast cancer is more likely to present with occult metastases than an older women, but the cancers themselves are inherently not more aggressive.

The mortality for black women exceeds that of white women at 20 years (41% versus 27%), the difference is due to a dramatic difference in early mortality rates (Fig. 6a,b) and to a lesser degree in hastening time to death (Fig. 6c).

## Part 2: Treatments effects: theory

The benefit of a treatment for breast cancer in terms of reducing mortality can be expressed in several ways. First, we hope for a reduction in the number of deaths. Most women who die will die within 20 years of diagnosis [1] and (although there are exceptions) for statistical purposes, 20-year survival without recurrence is a reasonable surrogate for cure [2]. However, in most studies, subjects are followed for fewer than 20 years and mortality benefit is typically measured in shorter terms, i.e. improvement in 5- and 10-year actuarial survival. Other commonly used indices to measure mortality reduction include hazard ratios (treated versus untreated), median survival times and time to death. There are various tabular and graphical representations of these metrics.

In the preceding section, we show that features of the cancer and the host (age and race) can impact on cancer mortality in different ways. We can also apply these measures to clarify the mode of action of breast cancer treatment. First, if a treatment eliminates all the cancer cells in the body (cytotoxic), the patient should be cured—i.e. if there are no residual cancer cells, none can flourish. We refer to this as ‘curability’. Second, a treatment may not eliminate all the cancer cells, but may shrink the tumour mass or slow tumour growth (cytostatic); for example, a treatment that does not kill cells but extends the doubling time of the surviving cancer cells will increase life expectancy even if the patient eventually dies of her cancer (progression delay). In the models presented here, we assume that if a woman is to die of her cancer she will do so in the 20-year period following diagnosis. A treatment may have both curative and anti-proliferative properties—the net benefit of such a treatment in a cohort of treated women will be the sum of benefits of cure and of progression delay.

## Cure versus progression delay: models

For the following hypothetical scenarios, we consider the basic model to recapitulate the survival experience of 45,647 ER-positive breast cancer patients diagnosed in the SEER database between 1990 and 1995 and then introduce two theoretical treatments. In this database, the actuarial 20-year breast cancer mortality was 72.4%. By simulation, we can evaluate how the effects of cytotoxic and cytostatic treatments are expected to influence the shape of the mortality curves. To illuminate the two models in terms of expected survival patterns, we have simulated cohorts of 91,294 women (45,647 treated and 45,647 not treated) under the two scenarios.

### Scenario 1: the cure model

**Fig. 9:**
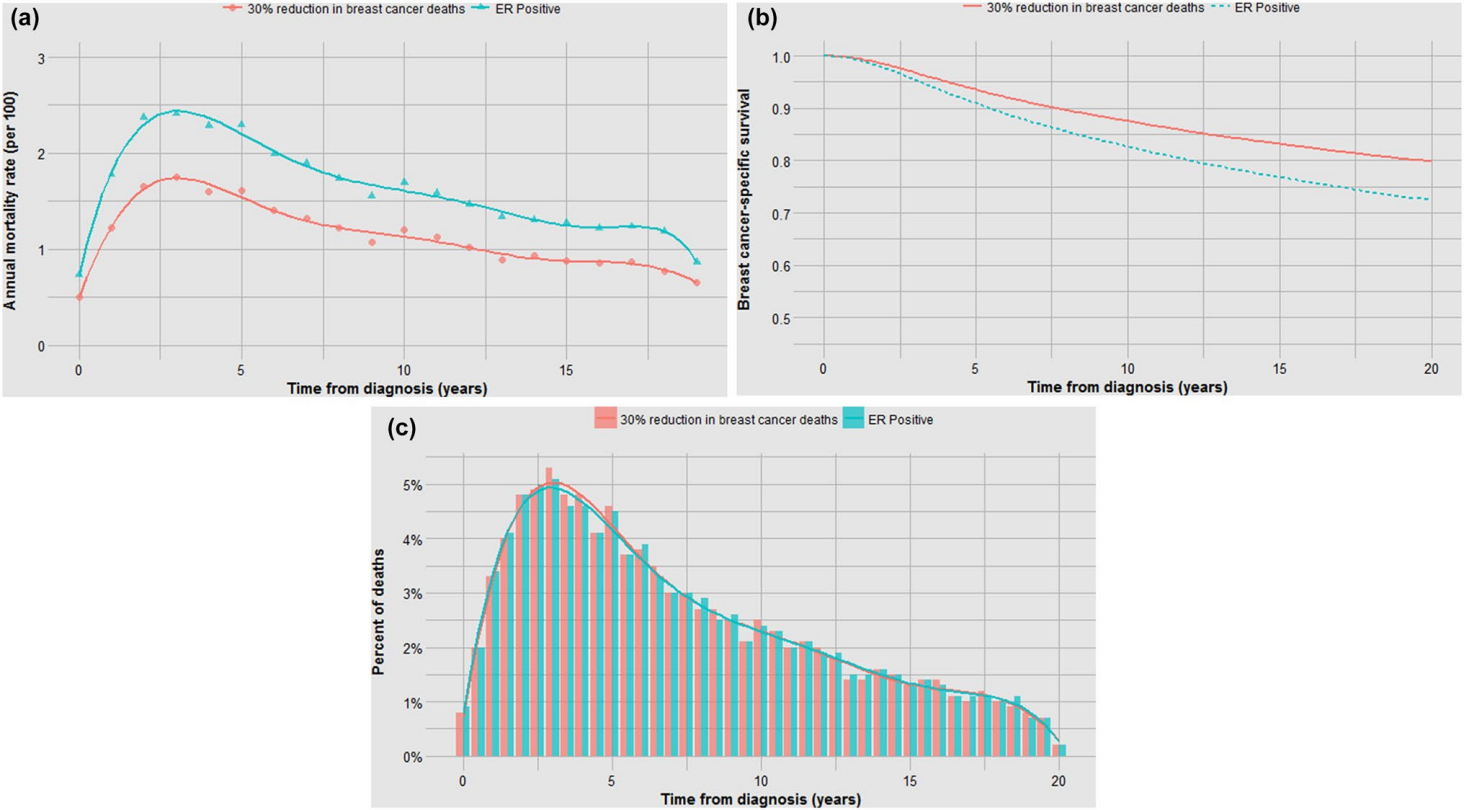
**a** Impact of 30% reduction in deaths on annual mortality rates, ER-positive patients in SEER. **b** Impact of 30% reduction in deaths on actuarial survival, ER-positive patients in SEER. **c** Impact of 30% reduction in deaths on time to death, ER-positive patients in SEER

We consider a cohort of 45,647 women with a survival of 72.4% at 20 years. Assume that a new treatment prevents 30% of all deaths (from 22.1 to 15.5%). In this simulation, we randomly removed 30% of deaths from the cohort and assumed that these women were alive at 20 years. We assume further that the time to death of women who are not cured is the same as in the absence of treatments. The three curves representing the survival experience of the untreated and treated women are presented in Fig. 9a–c.

### Scenario 2: the progression model

**Fig. 10:**
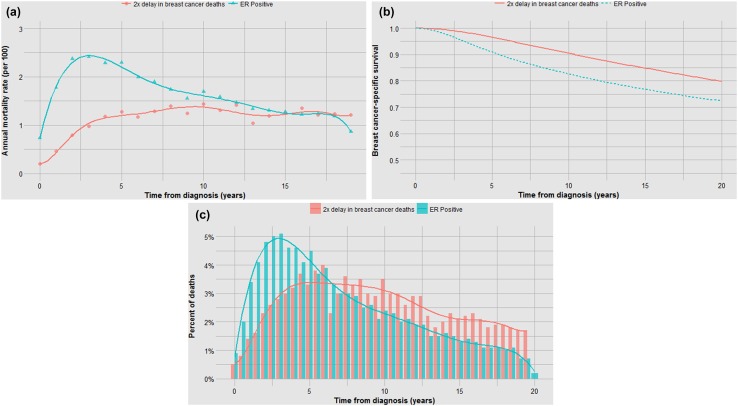
**a** Impact of doubling time to death on annual hazard rates, ER-positive patients in SEER. **b** Impact of doubling time to death on actuarial survival, ER-positive patients in SEER. **c** Impact of doubling time to death on time to death, ER-positive patients in SEER

We now consider a drug that does not cure patients but doubles the time to death for each patient. We have given the drug to a cohort of 45,647 women with the same inherent mortality risk as the untreated cohort in scenario 1. The net benefit in terms of survival at 20 years is from 72.4 to 79% (i.e. the same as in scenario 1). The three curves are presented in Fig. 10a–c. The intervention doubled the time to death for individual patients; this resulted in an increase in the mean time to death from 6.3 to 9.2 years (Table 2). These curves are notable in that a profound impact on delaying the time to death has a relatively modest impact on mortality; i.e. if we double the life expectancy of each patient in the study, we improve actuarial survival at 20 years from 72.4 to 79.9%. This is equivalent to curing 30% of the patients.

**Table 2:** Survival experience: all women with ER-positive breast cancer in SEER with simulated effects of two theoretical treatments

Group	*N*	Annual mortality rate (%)	% of Cohort dead of breast cancer	10 year actuarial survival (%)	20 year actuarial survival (%)	Median time to death [10th, 90th percentile]^a^	Mean time to death (years)^a^	% of Breast cancer deaths years 1–5^a^	% of Breast cancer deaths years 15–20^a^
ER positive	45,647	1.70	22.1	82.6	72.4	6.3 [1.9, 15.3]	7.5	39.3	11
30% mortality reduction	45,647	1.19	15.5	87.5	79.8	6.3 [1.9, 15.3]	7.5	39.5	11
Double time to death	45,647	1.09	15.4	90.3	79.9	9.2 [3.2, 17.3]	9.7	21.7	19.5

^a^In deceased patients

Note that although the survival benefit in both scenarios at 20 years is the same (80% vs. 72%) the actuarial survival curves appear different (Figs. 9b, 10b). Furthermore, in both scenarios progression to death appears to be delayed. Although the median survival was not reached, we can estimate the time to reach 85% actuarial survival. In scenario 1, the time required for 15% of the patients in the cohort to die is delayed by 3 years. In scenario 2, the time required for 15% of the patients in the cohort to die is delayed by 5 years. In actual fact, time to death is only altered in scenario 2. Statistically speaking, we use the term *progression delay* to describe the effect of a curative drug—i.e. we might report that the drug has increased the mean time to progression (or to death) from 6.3 years to 9.2 years—implying tumour growth is slowed (Table 2). The ‘delay in progression’ of 2.9 years is misleading because it does not accurately reflect the underlying tumour biology, i.e. tumour growth has not been slowed whatsoever—the entire effect is due to reducing the number of women at risk of dying at t_0_.

## Part 3: The banting database: tamoxifen

The simulated time-to-death curves under curative treatments and delaying death treatments are radically different (Figs. 9c, 10c). By comparing actual patient cohort data with the simulated data under the two models above (which represent cure and progression delay), we can ask which of the two scenarios best fits the empiric data and is therefore the most likely. Here we seek to determine by visual inspection to what extent the clinical benefit of a common treatment (tamoxifen) on mortality is dependent on the elimination of all cancer cells and to what extent the benefit is likely due to slowing the growth of persistent cancers.

**Fig. 11:**
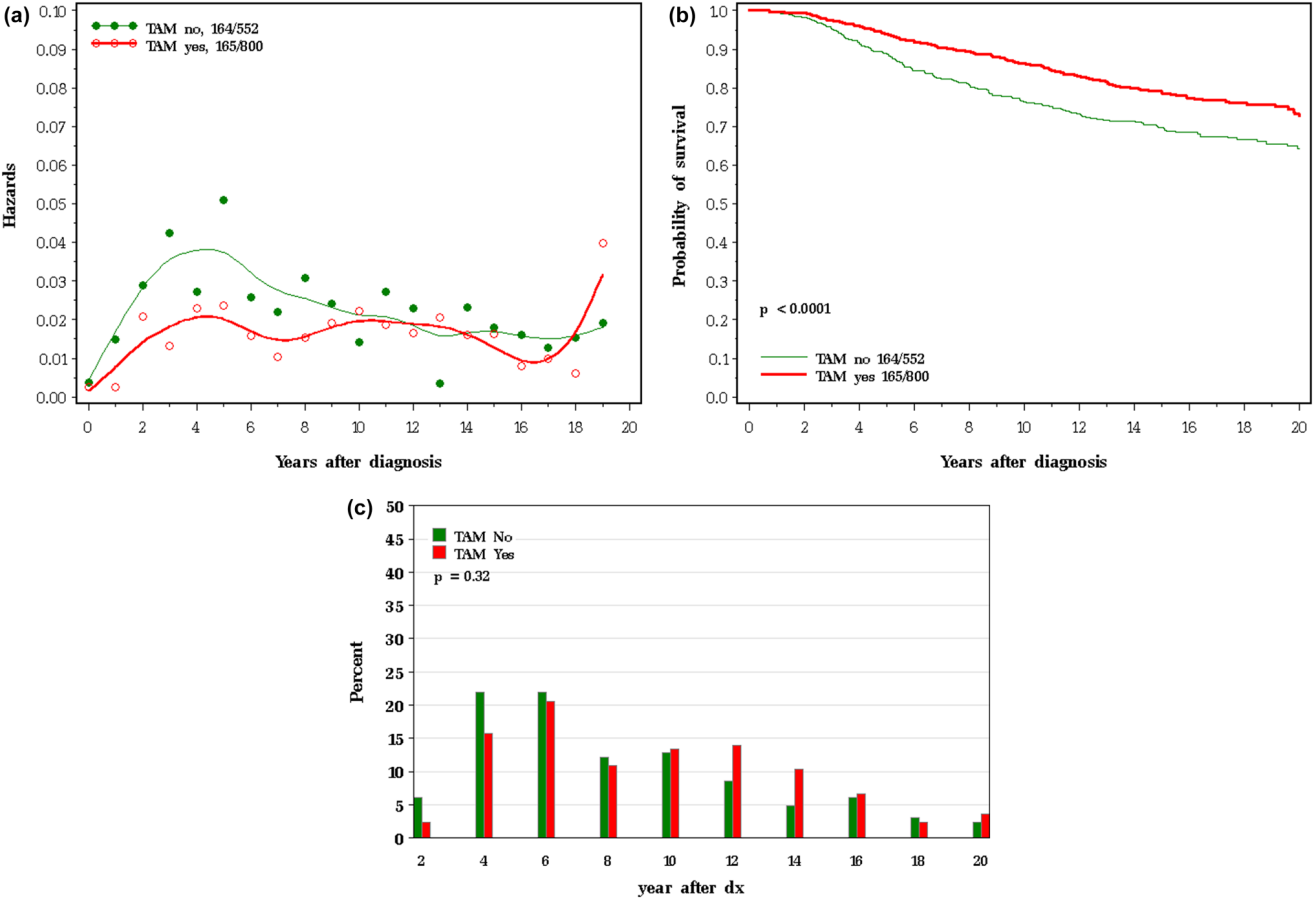
**a** Impact of tamoxifen therapy on annual mortality, ER-positive patients in Banting database. **b** Impact of tamoxifen therapy on actuarial survival, ER-positive patients in Banting database. **c** Impact of tamoxifen therapy on time to death, ER-positive patients in Banting database

We examined the actual mortality experience of women with breast cancer in the Henrietta Banting Breast Cancer database. In this database, 2305 women were diagnosed with primary invasive breast cancer (stages I–III) and were treated at Women’s College Hospital between 1987 and 2000. For each patient, we abstracted information on age at diagnosis, lymph node status, ER status, treatments received (chemotherapy, tamoxifen) and dates and causes of death. Patients were followed from the date of diagnosis to death from breast cancer, death from another cause or date of last follow-up. We then constructed mortality curves as presented above for the SEER database. Of the 1373 women with ER-positive breast cancer, 800 women took tamoxifen (59.2%) and 552 women did not take tamoxifen (40.8%). The mean years of use among tamoxifen users was 4.2 years. The survival experiences of the women with ER-positive breast cancers who did and who did not take tamoxifen are compared in Table 3 and Fig. 11a–c. The tamoxifen-treated women experienced an increase in time to death, suggesting that tamoxifen slows the growth of surviving cancers. In particular, the empirical data in Fig. 11a for tamoxifen-treated patients are very similar in appearance to the modelled data under a delayed progression model (Fig. 10a). In the simulated model, the median time to death was delayed by 2.9 years (6.3–9.2 years) (Table 2). In the data based on actual tamoxifen use, the time to death was delayed by an average of 2.0 years in users versus non-users (6.1–8.1 years).

**Table 3:** Survival experience: all women with ER-positive breast cancer in the Banting database

Group	*N*	Annual mortality rate (%)	% of Cohort dead of breast cancer	10-year actuarial survival (%)	20-year actuarial survival (%)	Median time to death [10th, 90th percentiles]^a^	Mean time to death (years)^a^	% of Breast cancer deaths years 1–5^a^	% of Breast cancer deaths years 15–20^a^
All ER positive	1,373	1.89	24.6	82.2	68.8	6.8 [2.6, 14.8]	6.8	32.5	8.6
Took tamoxifen	800	1.54	20.6	86.4	72.5	8.1 [2.8, 15.0]	8.1	28.5	9.1
Did not take tamoxifen	552	2.36	29.7	76.2	63.5	6.2 [2.5, 14.3]	6.1	36.0	7.9

^a^In deceased patients

## General discussion

The fate of a woman with breast cancer depends foremost on whether or not the breast cancer has metastasized at the time of diagnosis (metastatic potential). In the absence of metastases, local treatment is usually curative. For a small number of patients (5%), metastases will be clinically apparent at diagnosis (stage IV), but for most women with metastases, the metastases are subclinical at diagnosis and do not become apparent until later on (stages I–III) [1]. The second relevant factor is whether or not the prescribed treatment eradicates the latent metastases completely (curability). If all metastatic cells are eradicated, then there is no chance that these can proliferate. Assuming that all deaths take place within 20 years of diagnosis, the extent of curability can be approximated by the difference in actuarial survival at 20 years for treated versus untreated patients. The third relevant factor is the growth rate of the metastases over the lifespan of the patient (aggressivity). This is relevant for those women for whom the chemotherapy/hormonal therapy did not eradicate all of the cancer cells in the metastatic niche. The inherent growth rate is reflected in the time to death among patients who died. Each of the three factors contributes to prognosis, but they are also correlated; for example, tumour grade is associated with the presence of metastases at diagnosis and with tumour aggressivity (Fig. 4).

This analysis uncovered several interesting relationships. First, it appears that the relatively poor prognosis of women under 40 can be accounted for by the increased probability of cancer having latent metastases at diagnosis (Fig. 5). The benefit of chemotherapy appears to be independent of the age of the patient [3] and among women who die of their breast cancer, age of diagnosis is not predictive of time to death. It is not clear from this study if the high proportion of cancers that are metastatic at diagnosis is a consequence of other adverse factors associated with young age or if age has an independent effect beyond grade, size and nodal status. This will be a topic of a future study. In contrast, black women fared worse than white women both because of a higher probability of latent metastases and a relatively rapid time to death (Fig. 6). Tumour grade is highly predictive of mortality because it is associated with both the probability of metastases and the time to death (Fig. 4). Because race and grade are strongly correlated with both metastatic potential and with aggressivity, the Kaplan–Meier curves are widely separated for these two factors (Figs. 4b, 6b). In a previous study of the Banting database, we noted that the time to death was relatively rapid for women with triple-negative cancers versus ER-positive cancers, in particular because a high proportion of women with triple-negative cancers died in the first 5 years following diagnosis [4]. In the present paper, we show that this is a general phenomenon; that is, a high proportion of early deaths is a property of all breast cancers with high mortality rate. This is not merely because of a diminishing denominator. As can be seen in Table 1 (supplemental), the annual hazard rates decline with time from diagnosis for ER-negative cancers but the annual mortality rates for ER-positive cancers are relatively stable over 20 years, in particular for women who took tamoxifen. The same inflection point in mortality at roughly 5 years is seen for women with high mortality because of black race, high grade, young age and positive nodal status. The underlying basis for this phenomenon is a matter of future study.

The relative stability in annual hazard rates over the 20-year period for women with ER-positive, low-grade, small or node-negative cancers indicates that in clinical studies of low-risk cancers it will be necessary to follow for a longer time than 10 years to adequately capture cumulative mortality.

There are several limitations of our approach. We focused on a group of breast cancer patients who had a minimum of 20 years of follow-up but there some patients will die of breast cancer beyond this point. However, there are few databases available which follow women for 30 or 25 years. Our study was based on two observational cohorts and we have not considered other covariates when individual prognostic factors are analysed. In the case of the Banting data set, there was limited power for some subgroup comparisons.

## Electronic supplementary material

Below is the link to the electronic supplementary material.

Supplementary material 1 (DOCX 15 KB)
