# Supplementary material for: Time to death in breast cancer patients as an indicator of treatment response
Source: Breast Cancer Res Treat. 2018 Aug 30;172(3):659–69. doi: 10.1007/s10549-018-4935-3 (PMC6245100; doi:10.1007/s10549-018-4935-3)
Supplement: Supplementary file 1 — Supplementary material 1 (DOCX 15 KB) [file 10549_2018_4935_MOESM1_ESM.docx]

**Supplementary Table 1.** Annual hazard rates, patients with ER-positive or ER-negative cancer in SEER

| Year | ER-positive | | | ER-negative | | |
| --- | --- | --- | --- | --- | --- | --- |
|  | Number at risk | Number of deaths | Annual mortality rate (%) | Number at risk | Number of deaths | Annual mortality rate (%) |
| 0 - 1 | 45,647 | 334 | 0.74 | 14,759 | 454 | 3.12 |
| 1 - 2 | 44,661 | 781 | 1.78 | 14,149 | 1,003 | 7.37 |
| 2 - 3 | 43,118 | 1,002 | 2.37 | 12,938 | 885 | 7.12 |
| 3 - 4 | 41,175 | 971 | 2.41 | 11,843 | 591 | 5.16 |
| 4 - 5 | 39,202 | 878 | 2.29 | 11,036 | 431 | 4.02 |
| 5 - 6 | 37,320 | 837 | 2.30 | 10,397 | 295 | 2.90 |
| 6 - 7 | 35,443 | 689 | 1.99 | 9,927 | 227 | 2.34 |
| 7 - 8 | 33,685 | 626 | 1.90 | 9,491 | 148 | 1.59 |
| 8 - 9 | 32,002 | 543 | 1.74 | 9,144 | 106 | 1.18 |
| 9 - 10 | 30,344 | 461 | 1.56 | 8,857 | 82 | 0.94 |
| 10 - 11 | 28,771 | 475 | 1.70 | 8,588 | 88 | 1.04 |
| 11 - 12 | 27,174 | 421 | 1.59 | 8,313 | 75 | 0.92 |
| 12 - 13 | 25,665 | 368 | 1.47 | 8,018 | 80 | 1.02 |
| 13 - 14 | 24,229 | 317 | 1.34 | 7,718 | 61 | 0.80 |
| 14 - 15 | 22,904 | 292 | 1.31 | 7,449 | 55 | 0.75 |
| 15 - 16 | 21,597 | 268 | 1.27 | 7,180 | 42 | 0.59 |
| 16 - 17 | 20,322 | 241 | 1.22 | 6,930 | 55 | 0.81 |
| 17 - 18 | 19,081 | 229 | 1.24 | 6,640 | 37 | 0.57 |
| 18 - 19 | 17,898 | 206 | 1.19 | 6,402 | 30 | 0.48 |
| All |  | 10,080 | 1.70 |  | 4,777 | 2.63 |
